# The relationship of reproductive factors with adiposity and body shape indices changes overtime: findings from a community-based study

**DOI:** 10.1186/s12967-023-04000-1

**Published:** 2023-02-22

**Authors:** Mina Amiri, Maryam Mousavi, Fereidoun Azizi, Fahimeh Ramezani Tehrani

**Affiliations:** 1 Reproductive Endocrinology Research Center, Research Institute for Endocrine Sciences, Shahid Beheshti University of Medical Sciences, 24 Parvaneh, Yaman Street, Velenjak, P. O. Box 19395-4763, 1985717413 Tehran, I. R. of Iran; 2 Department of Biostatistics, Faculty of Medical Sciences, Tarbiat Modares University, Tehran, Iran; 3 Endocrine Research Center, Research Institute for Endocrine Sciences, Shahid Beheshti University of Medical Sciences, Tehran, I. R. of Iran

**Keywords:** Adiposity, Body mass index (BMI), Waist circumference (WC), A body shape index (ABSI), Age at menarche, Age at menopause, Pregnancy

## Abstract

**Background:**

Studies focusing on the relationships of adiposity and body shape indices with reproductive factors have reported conflicting results. This study aimed to investigate the influence of reproductive factors on adiposity and body shape indices changes overtime.

**Materials and methods:**

In this community-based prospective study, 1636 postmenopausal women were selected from Tehran Lipid and Glucose Study (TLGS). The unadjusted and adjusted Generalized Estimating Equation models (GEE) were applied to investigate secular longitudinal trends of adiposity and body shape indices.

**Results:**

According to the adjusted GEE models, mean changes in body mass index (BMI) in women with early menarche was 1.18 kg/m^2^ higher than those with normal menarche age (P = 0.030). Moreover, the mean changes in BMI overtime were 0.11 kg/m^2^ higher in women with premature/early menopausal age than those with normal menopausal age (P = 0.012). Mean changes of waist circumference (WC) in women with late menopause were 2.27 cm higher than those with normal menopausal age (P = 0.036). We also observed higher mean changes in a body shape index (ABSI) in women with late menopause (P = 0.037), compared to those with normal menopausal age. We found a marginal effect of parity on BMI and WC as well.

**Conclusions:**

This study demonstrated higher BMI in females with earlier menarche age. We also showed higher values of BMI overtime in women with premature/ early menopause, whereas women with late menopausal age had higher WC and ABSI values. However, more longitudinal studies investigating body composition indices by adjusting all potential confounders are still required to confirm our study findings.

## Background

Adipose tissue disorders, such as obesity and central obesity, are increasing worldwide and are associated with an increased risk of adverse health outcomes, in particular cardio-metabolic disturbances [1, 2], leading to an increase in all-cause mortality [3].

Epidemiologic studies demonstrate that in most populations, the prevalence of adipose tissue disturbances is greater in women than in men [4, 5]. Besides, women have a greater percentage of body fat to prepare for child-bearing and body composition during their reproductive cycle [6] and experience more dissatisfaction with their body shape [7]. These gender differences highlight the role of women’s reproductive characteristics in the formation of the body shape and variations related to adiposity and its indices [6–8]. There is evidence demonstrating that waist circumference (WC) and waist-to-hip ratio (WHR) are associated with sex hormone-binding globulin (SHBG) and sex hormones such as free estradiol and free testosterone independently of body mass index (BMI). As a result, female sex hormones may regard as one of the main factors affecting fat distribution [9]. Increased concentrations of estrogens or other reproductive hormones during puberty are differentially associated with the activation of the homeobox family (HOX) and other genes to determine regional adipose distribution [10]. Hormonal changes during pregnancy can be also associated with remaining adiposity after pregnancy [11–13]. There is evidence demonstrating that multiparous women have higher BMI than their nulliparous counterparts [14]. It is also documented that women’s body shape can be affected by their pregnancy status [15]. Previous studies on both populations of pre-and postmenopausal women also highlight the role of the hormonal environment in creating changes related to body fatness, indicating that the rate of reproductive aging is significantly associated with the body fat pattern. It has been shown BMI of women with abnormal levels of sex hormones is more correlated with their hormonal status than their age or menopausal status [16].

Although numerous studies have evaluated the relationships of adiposity and body shape indices with reproductive factors like age at menarche, menopausal age, and pregnancy history, their results are still conflicting and inconclusive [6, 14–29]. Moreover, these associations have been complicated by reverse causation since most available studies had cross-sectional designs and could not demonstrate causal relationships [6, 14–20]. Furthermore, the majority of studies have assessed some adiposity indices like BMI, waist circumference (WC), and WHR and a limited number of studies have specifically evaluated body shape concerning reproductive factors [15, 19, 30]. This community-based prospective study, therefore, aimed to investigate the influence of reproductive history on the adiposity and body shape indices changes overtime after adjusting potential confounders.

## Materials and methods

### Study design and participants

For this population-based prospective study, participants were selected from among participants of the Tehran Lipid and Glucose Study (TLGS). TLGS is an ongoing prospective cohort initiated in 1998, in which 15,005 participants aged ≥ 3 years were assessed [31]. In summary, information on various risk factors for non-communicable diseases, demographic variables, and reproductive histories was collected during face-to-face interviews conducted every 3 years in 6 follow-up visits. Among TLGS participants, we included all women aged ≥ 20 years who participated in the baseline and at least one follow-up whose reproductive and menopause status was defined. Out of 5374 postmenopausal women who entered at baseline, 1191 women were excluded due to the missing menarche age and 2547 women due to the missing menopausal age. Finally, a total of 1636 women were eligible and analyzes for the present study.

### Measurements

All study participants were interviewed to obtain medical, obstetrics, and family histories using pretested questionnaires [32]. Clinical and anthropometric measurements were assessed by trained examiners at each follow-up, details of which have been previously published [33]. In summary, weight was measured when they were minimally clothed using a digital scale (Seca 707, Seca GmbH) and rounded to the nearest 100 g. Height was measured without shoes in the standing position with shoulders in normal alignment, using a tape measure. Waist circumference was measured with an unstretched tape measure at the level of the umbilicus without any pressure on the body surface and recorded to the nearest 0.1 cm. Hip circumference was measured at the level of the anterior superior iliac spine without any pressure on the body surface.

### Term definition

Body mass index (BMI) was calculated as weight in kilograms (kg) divided by height squared (m2). A body mass index (ABSI) was calculated based on the following formula: [WC (cm)/[BMI ^2/3^ × height (m)^1/2^] [34]. Smoking status was classified into two categories, including ever smokers (current users and those who used to smoke in the past) and never smokers. For evaluating physical activity, a modified activity questionnaire (MAQ) was used, which is evaluated and validated in the Iranian population. According to the questionnaire, physical activity has been specified as low (MET < 600 min/wk), moderate (MET 600–1499 min/wk), and high (MET ≥ 1500 min/wk) levels [35, 36].

Age at menarche was defined as the age at the first menstrual bleeding. Age at menarche < 11 years, 12–15 years, and ≥ 16 were considered as early, normal, and late menarche, respectively. According to the World Health Organization classification, menopause was defined as the absence of spontaneous menstrual bleeding for more than 12 months, for which no other pathologic or physiologic cause could be determined [37]. Age at natural menopause was defined as younger than 40 years (premature menopause), 40–44 years (early menopause), 45–54 years (reference category), and 55 years or older (late menopause) [38, 39].

### Statistical analysis

The baseline characteristics of participants are described by a median, interquartile range (IQR) in non-normal continuous variables, and in cases of non-rejection of the normality assumption, mean (standard deviation) was used. For evaluating the normality hypothesis, we conducted the Kolmogorov–Smirnov normality test. The categorical variables were described as frequencies (%). Statistical analysis was performed using Generalized Estimating Equation models (GEE) to investigate the secular longitudinal trends of adiposity and body shape indices including BMI, waist, and ABSI and evaluate the effect of reproductive factors on these trends. We used z-scores of ABSI due to the small values of this variable.

The GEE analysis accounts for correlations within subjects through a working correlation matrix and enables researchers to accurately estimate the effect size in case of incomplete data (missing variables in some repeated measures), which is common in cohort studies. We assessed the time trends of each adiposity index by fitting unadjusted and adjusted GEE models (by adjusting for baseline age, parity, smoking, education, and physical activity status).

All statistical analysis was performed in SPSS16 and STATA (version 12; STATA Inc., College Station, TX, USA). The p-values less than 0.05 were considered statistically significant.

## Results

In this study with a median (IQR) of 16 (15–17) follow-up years, a total of 1636 eligible women were analyzed. Table 1 shows the baseline characteristics of the study participants. The median (IQR) of age and BMI of the study participants were 57.0 (52.0–63.0) years and 29.6 (26.7–32.8) Kg/m^2^, respectively. Moreover, the median (IQR) of age at menarche and menopause were 14 (13–15) years and 49 (45–52) years, respectively. The majority of participants had 6–12 years of education and a low level of physical activity.

**Table 1:** Baseline characteristics of the study participants

Variables	Total N = 1636
Baseline age^a^	57 (52–63)
BMI at baseline, Median (IQR)	29.62 (26.70–32.77)
Waist circumference at baseline, Median (IQR)	96 (88–103)
ABSI in baseline, Median (IQR)	0.08 (0.07–0.08)
Educational level (years)^b^	
< 6	538 (32.9)
6–12	1058 (64.7)
> 12	40 (2.4)
Smoking^b^	
Never	1539 (94.1)
Ever	97 (5.9)
Physical activity^b,c^	
Low	1135 (69.4)
Moderate to high	501 (30.6)
Age at menarche^a,d^	14 (13–15)
Early menarche^b^	92 (5.6)
Normal menarche^b^	1345 (82.2)
Late menarche^b^	199 (12.2)
Age at menopause^a,e^	49 (45–52)
Premature/early menopause^b^	463 (28.3)
Normal menopausal age^b^	1029 (62.9)
Late at menopausal age^b^	144 (8.8)
Length of reproductive^a^	35 (31–39)
Gravidity^a^	5 (3–7)
Parity^a^ Nulliparous^b^	5 (3–7)
0	74 (4.5)
1–2	268 (16.4)
≥ 3	1294 (79.1)
History of abortion^b^	
No	1014 (62)
Yes	622 (38)

Length of reproduction is the interval duration between age at menarche and age at menopause

*BMI* body mass index; *ABSI* a body shape index

^a^Median (interquartile range)

^b^Number (%)

^c^Physical activity has been specified as low (MET < 600 min/week), moderate (MET 600–1499 min/week), and high (MET ≥ 1500 min/week) levels

^d^Age at menarche < 11 years, 12–15 years, and ≥ 16 were considered as early, normal, and late menarche, respectively

^e^Age at natural menopause was defined as younger than 40 years (premature menopause), 40–44 years (early menopause), 45–54 years (reference category), and 55 years or older (late menopause)

**Fig. 1:**
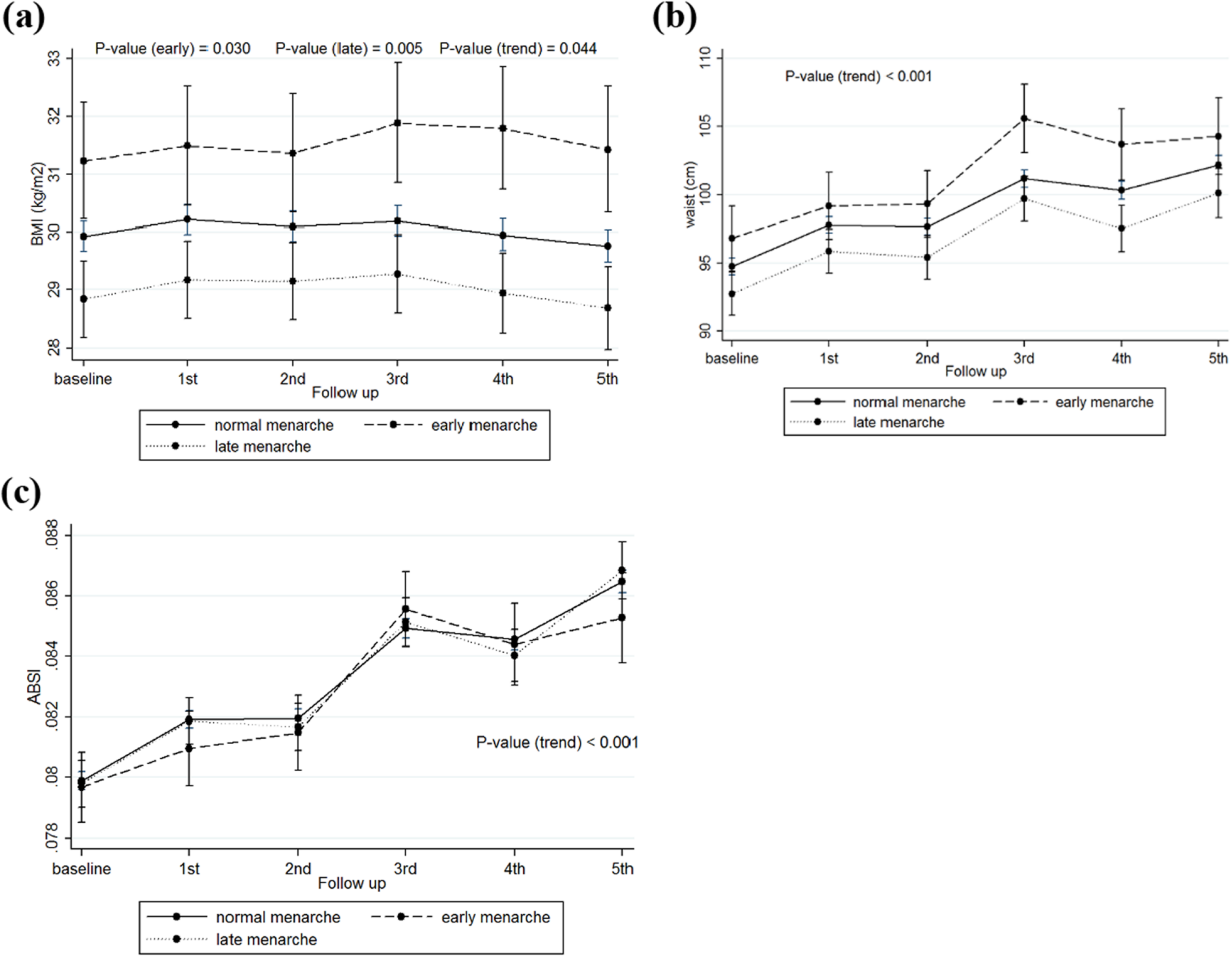
Trends of adiposity indices overtime based on age at menarche. **a** BMI trends based on the age at menarche subgroups; **b** Waist circumference trends based on the age at menarche subgroups; **c** ABSI trends based on the age at menarche subgroups. *BMI* body mass index; *ABSI* a body shape index

**Fig. 2:**
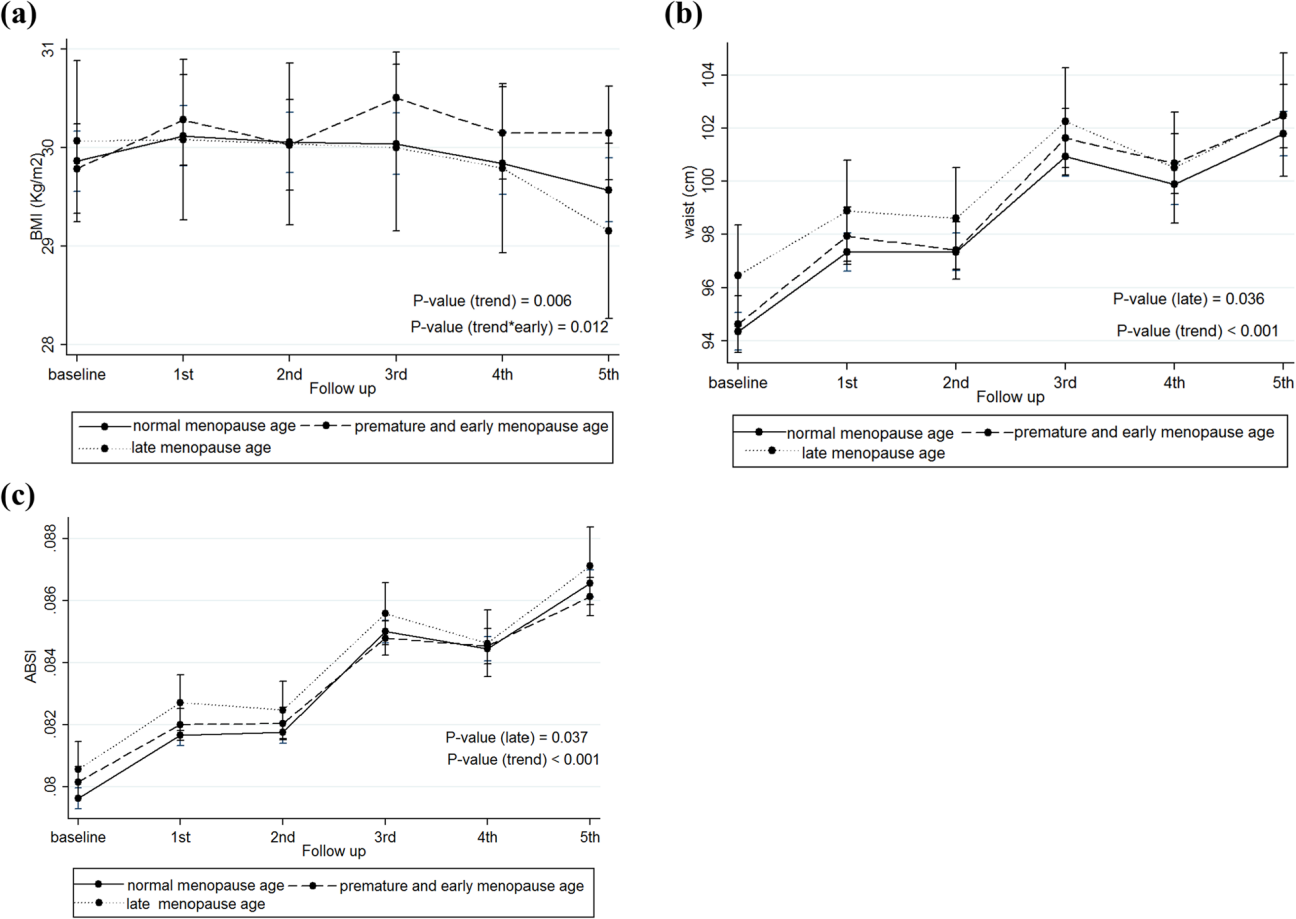
Trends of adiposity indices overtime based on the age at menopause. **a** BMI trends based on the age at menopause subgroups; **b** Waist circumference trends based on the age at menopause subgroups; **c** ABSI trends based on the age at menopause subgroups. *BMI* body mass index; *ABSI* a body shape index

**Fig. 3:**
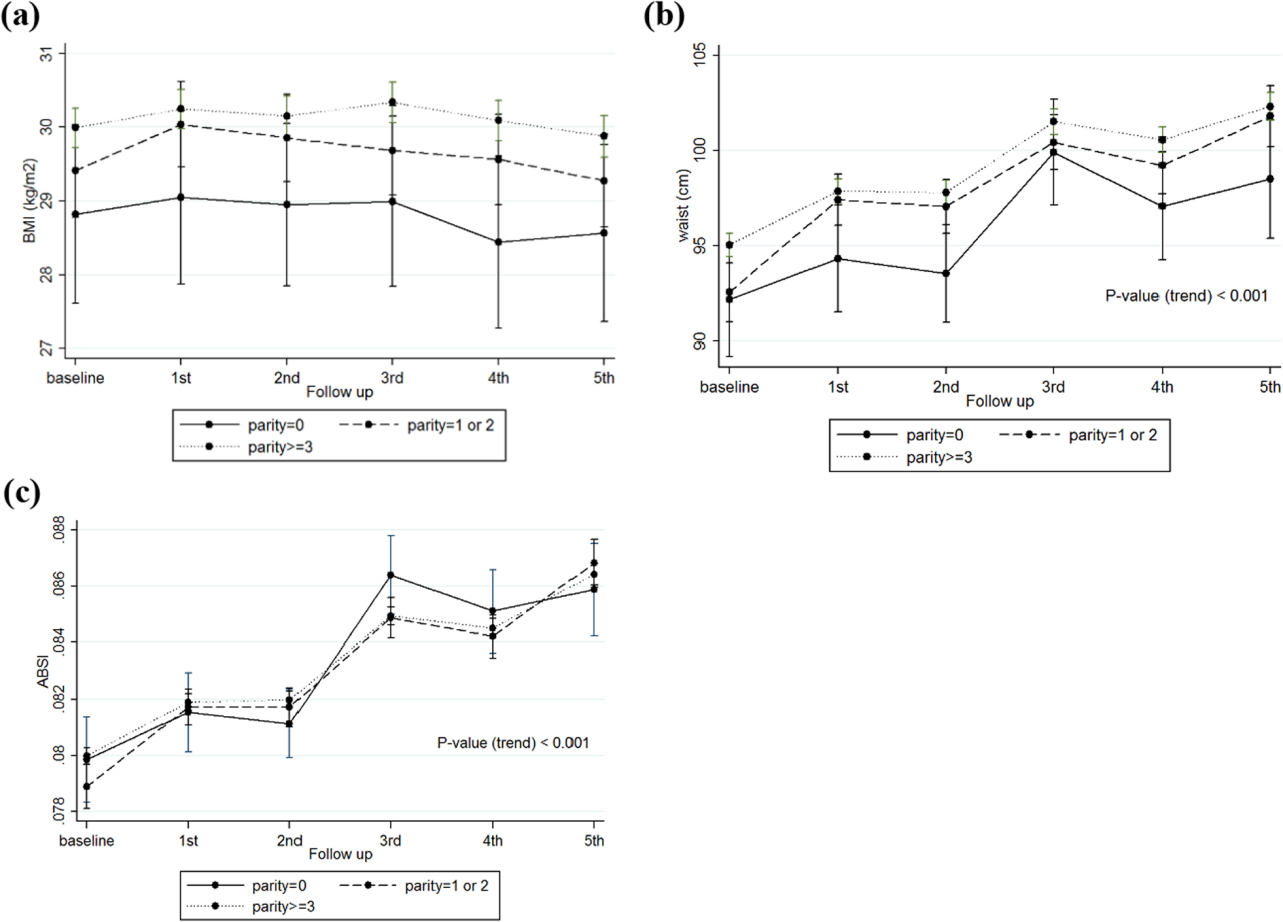
Trends of adiposity indices overtime based on the parity status. **a** BMI trends based on the parity subgroups; **b** Waist trends based on the parity subgroups; **c** ABSI trends based on parity subgroups. *BMI* body mass index; *ABSI* a body shape index

**Fig. 4:**
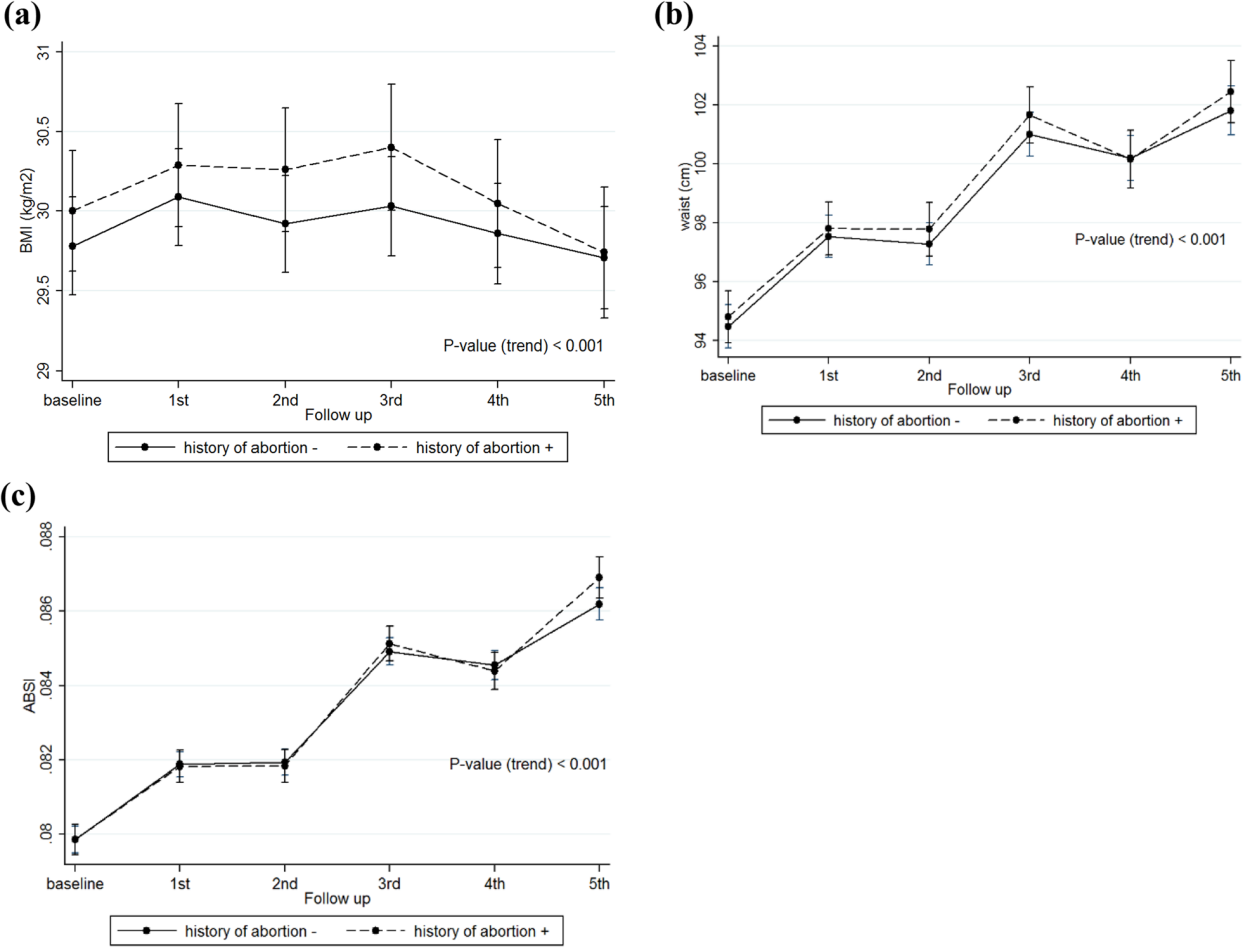
Trends of adiposity indices overtime based on the abortion status. **a** BMI trends based on the abortion subgroups; **b** Waist circumference trends based on the abortion subgroups, **c** ABSI trends based on the abortion subgroups. *BMI* body mass index; *ABSI* a body shape index

Tables 2–4 show the results of GEE models to estimate the effect of reproductive characteristics (age at menarche, age at menopause, parity, and abortion) on the trend of adiposity indices, i.e. BMI, WC, and ABSI with and without adjustment for age, educational level, smoking status, and physical activity. Figures 1, 2, 3, 4 illustrate trends of adiposity indices overtime in the different subgroups of the study participants based on their reproductive factors as well.

**Table 2:** Effects of menarche age on adiposity indices of participants over time

Reproductive factors	Outcomes	Model 1	P-value ^c^	Model 2	P-value ^c^
		Coefficient (95% CI)		Coefficient (95% CI)	
Menarche age^a^ (ref: Normal)	BMI (kg/m^2^)				
	Early	1.43 (0.35, 2.50)	**0.009**	1.18 (0.11, 2.25)	**0.030**
	Late	− 1.00 (− 1.75, − 0.25)	**0.008**	− 1.05 (− 1.79, − 0.31)	**0.005**
	Time	− 0.05 (− 0.09, − 0.006)	**0.024**	− 0.04 (− 0.09, − 0.001)	**0.044**
	Time × early	0.08 (− 0.09, − 0.26)	0.343	0.09 (− 0.09, 0.26)	0.327
	Time × late	0.004 (− 0.11,0.12)	0.946	0.006 (− 0.11, 0.12)	0.922
	Waist circumference (cm)				
	Early	1.45 (− 1.17, 4.07)	0.278	1.66 (− 0.94, 4.26)	0.211
	Late	− 1.66 (− 3.46, 0.14)	0.070	− 1.72 (− 3.50, 0.07)	0.059
	Time	1.32 (1.19, 1.45)	** < 0.001**	1.33 (1.20, 1.46)	** < 0.001**
	Time × early	0.26 (− 0.26, 0.79)	0.316	0.28 (− 0.24, 0.80)	0.295
	Time × late	− 0.08 (− 0.43, 0.27)	0.647	− 0.08 (− 0.42, 0.27)	0.664
	ABSI^b^				
	Early	− 0.19 (− 0.43, 0.04)	0.111	− 0.05 (− 0.28, 0.17)	0.640
	Late	− 0.02 (− 0.18, 0.15)	0.846	− 0.02 (− 0.17, 0.14)	0.829
	Time	0.20 (0.19, 0.21)	** < 0.001**	0.21 (0.20, 0.22)	** < 0.001**
	Time × early	− 0.003 (− 0.06, 0.05)	0.929	− 0.0004 (− 0.05, 0.05)	0.988
	Time × late	0.001 (− 0.04, 0.04)	0.955	0.003 (− 0.03, 0.04)	0.860

*BMI* Body mass index; *ABSI* A body shape index; *Ref* Reference; *CI* Confidence interval

Model 1: Unadjusted model

Model 2: Adjusted for baseline age, smoking, education, and physical activity status

Time variable means follow-up visits

^a^Age at menarche < 11 years, 12–15 years, and ≥ 16 were considered as early, normal, and late menarche, respectively

^b^The results of ABSI are presented by z-scores

^c^Significant p-values (p < 0.05) are bolded

### Age at menarche

The mean changes of BMI in women with early menarche were 1.43 kg/m^2^ higher than those with normal menarche age (95% CI 0.35, 2.50; P = 0.009), finding that remained significant even after adjustment for confounders (model 2) (1.18 kg/m2; 95% CI 0.11, 2.25; P = 0.030). On the other hand, the mean changes of BMI in women with late menarche were significantly lower than those with normal age at menarche in both models of unadjusted (− 1.00 kg/m^2^; 95% CI -1.75, − 0.25; P = 0.008) and adjusted (− 1.05 kg/m^2^; 95% CI − 1.79, − 0.31; P = 0.005). While BMI had uprising trends in all groups, regardless of their menarche age, the interaction between time and different subgroups of menarche age (early and late) was not significant. We also found a marginally significant effect of menarche age on WC, indicating that the mean changes of WC in women with late menarche age were lower than those with normal menarche age (− 1.66 cm; 95% CI − 3.46, 0.14; P = 0.070); this marginal effect remained after adjusting for potential confounders (− 1.72 cm; 95% CI − 3.50, 0.07; P = 0.059). We found no significant association between menarche age and ABSI overtime (Table 2).

### Age at menopause

This study revealed that the interaction effect of time and early menopausal age on mean changes in BMI was significant (0.10 kg/m^2^; 95% CI 0.02, 0.19; P = 0.017), indicating that mean changes in BMI overtime was 0.1 kg/m^2^ higher in women with premature/ early menopausal age than those with normal menopausal age, finding that remained significant even after adjusting confounders (0.11 kg/m^2^; 95% CI 0.02, 0.19; P = 0.012). This study also showed that mean changes of WC in women with late menopause were significantly higher than those with normal menopausal age (2.31 cm; 95% CI 0.19, 4.43; P = 0.033), finding that remained significant even after adjusting confounders (2.27 cm; 95% CI 0.15, 4.39; P = 0.036). We also observed higher mean changes of z-scores of ABSI in women with late menopause (0.37; 95% CI 0.18, 0.56; P < 0.001), compared to those with normal menopausal age, finding that remained significant even adjusting confounders (0.19; 95% CI 0.01, 0.38; P < 0.037). After adjusting confounders, the interaction effect of time and premature/ early menopause on mean changes of ABSI z-scores was significant (− 0.03; 95% CI − 0.05, − 0.0009; P = 0.043) (Table 3).

**Table 3:** Effects of menopausal age on adiposity indices in women of the TLGS over time

Reproductive factors	Outcomes	Model 1	P-value ^c^	Model 2	P-value ^c^
		Coefficient (95% CI)		Coefficient (95% CI)	
Menopause age^a^ (ref: Normal)	BMI (kg/m^2^)				
	Premature/early	0.37 (− 0.18, 0.92)	0.188	− 0.10 (− 0.67, 0.46)	0.722
	Late	− 0.13 (− 1.01, 0.75)	0.773	0.26 (− 0.62, 1.14)	0.566
	Time (year)	− 0.07 (− 0.12, − 0.02)	**0.005**	− 0.07 (− 0.11, − 0.02)	**0.006**
	Time × premature/early	0.10 (0.02, 0.19)	**0.017**	0.11 (0.02, 0.19)	**0.012**
	Time × late	− 0.10 (− 0.24, 0.04)	0.166	− 0.10 (− 0.24, 0.04)	0.163
	Waist circumference (cm)				
	Premature/early	0.24 (− 1.10, 1.57)	0.723	0.26 (− 1.01, 1.63)	0.703
	Late	2.31 (0.19, 4.43)	**0.033**	2.27 (0.15, 4.39)	**0.036**
	Time	1.33 (1.18, 1.47)	** < 0.001**	1.34 (1.19, 1.48)	** < 0.001**
	Time × premature/early	0.07 (− 0.19, 0.32)	0.611	0.06 (− 0.19, 0.31)	0.643
	Time × late	− 0.27 (− 0.70, 0.15)	0.209	− 0.28 (− 0.70, 0.15)	0.200
	ABSI^b^				
	Early	− 0.10 (− 0.22, 0.02)	0.093	0.11 (− 0.007, 0.23)	0.066
	Late	0.37 (0.18, 0.56)	** < 0.001**	0.19 (0.01, 0.38)	**0.037**
	Time	0.21 (0.20, 0.23)	** < 0.001**	0.22 (0.20, 0.24)	** < 0.001**
	Time × premature/early	− 0.03 (− 0.05, − 0.0004)	**0.047**	− 0.03 (− 0.05, − 0.0009)	**0.043**
	Time × late	− 0.02 (− 0.06, 0.03)	0.390	− 0.02 (− 0.07, 0.02)	0.308

*BMI* Body mass index; *ABSI* A body shape index; *Ref* Reference; *CI* Confidence interval

Model 1: Unadjusted model

Model 2: Adjusted for baseline age, smoking, education, and physical activity status

Time variable means follow-up visits

^a^Age at natural menopause was defined as younger than 40 years (premature menopause), 40–44 years (early menopause), 45–54 years (reference category), and 55 years or older (late menopause)

^b^The results of ABSI are presented by z-scores

^c^Significant p-values (p < 0.05) are bolded

### Parity and abortion

We also found a marginal effect of parity on BMI and WC, indicating that women with parity ≥ 3 had higher mean changes in these parameters, compared to those with less parity. Abortion was not significantly associated with none of the adiposity indices (Table 4).

**Table 4:** Effects of parity and abortion on adiposity indices in women of the TLGS over time

Reproductive factors	Outcomes	Model 1	P-value ^b^	Model 2	P-value ^b^
		Coefficient (95% CI)		Coefficient (95% CI)	
Parity (ref: 0)	BMI (kg/m^2^)				
	1–2	0.74 (− 0.63, 2.10)	0.290	0.83 (− 0.51, 2.18)	0.228
	≥ 3	1 (− 0.25, 2.24)	0.117	1.06 (− 0.17, 2.30)	0.092
	Time	− 0.09 (− 0.29, 0.11)	0.380	− 0.08 (− 0.28, 0.12)	0.413
	Time × (1–2)	− 0.003 (− 0.22, 0.22)	0.982	− 0.003 (− 0.22, 0.22)	0.978
	Time × (≥ 3)	0.06 (− 0.15, 0.26)	0.595	0.05 (− 0.15, 0.26)	0.607
	Waist circumference (cm)				
	1–2	1.67 (− 1.72, 5.06)	0.335	1.75 (− 1.61, 5.11)	0.307
	≥ 3	3.09 (− 0.008, 6.19)	0.051	2.84 (− 0.23, 5.92)	0.070
	Time	1.18 (0.58, 1.79)	** < 0.001**	1.20 (0.60, 1.80)	** < 0.001**
	Time × (1–2)	0.19 (− 0.48, 0.87)	0.573	1.19 (− 0.48, 0.86)	0.577
	Time × (≥ 3)	0.15 (− 0.47, 0.77)	0.644	1.13 (− 0.48, 0.75)	0.673
	ABSI^a^				
	1–2	− 0.03 (− 0.35, 0.28)	0.849	− 0.05 (− 0.35, 0.25)	0.749
	≥ 3	0.14 (− 0.15, 0.43)	0.346	0.06 (− 0.21, 0.34)	0.660
	Time	0.21 (0.15, 0.28)	** < 0.001**	0.22 (0.16, 0.28)	** < 0.001**
	Time × (1–2)	0.01 (− 0.06, 0.08)	0.778	0.01 (− 0.06, 0.08)	0.785
	Time × (≥ 3)	− 0.01 (− 0.08, 0.05)	0.666	− 0.01 (− 0.08, 0.05)	0.674
Abortion (ref: No)	BMI (kg/m^2^)				
	Abortion	0.35 (− 0.15, 0.85)	0.169	0.25 (− 0.25, 0.75)	0.319
	Time	− 0.04 (− 0.09, 0.01)	0.127	− 0.03 (− 0.08, 0.01)	0.165
	Time × abortion	− 0.02 (− 0.1, 0.05)	0.575	− 0.02 (− 0.09, 0.06)	0.651
	Waist circumference (cm)				
	Abortion	0.41 (− 0.79, 1.61)	0.502	0.19 (− 1.03, 1.37)	0.783
	Time	1.30 (1.15, 1.45)	** < 0.001**	1.31 (1.16, 1.46)	** < 0.001**
	Time × abortion	0.07 (− 0.17, 0.30)	0.572	0.1 (− 0.17, 0.29	0.618
	ABSI^a^				
	Abortion	− 0.02 (− 0.13, 0.09)	0.716	− 0.04 (− 0.14, 0.07)	0.481
	Time	0.19 (0.18, 0.21)	** < 0.001**	0.20 (0.19, 0.22)	** < 0.001**
	Time × abortion	0.02 (− 0.007, 0.04)	0.160	0.02 (− 0.009, 0.04)	0.208

BMI: Body mass index; ABSI: A body shape index; Ref: Reference; CI: Confidence interval

Model 1: Unadjusted model

Model 2: Adjusted for baseline age, smoking, education, and physical activity status

Time variable means follow-up visits

^a^The results of ABSI are presented by z-scores

^b^Significant p-values (p < 0.05) are bolded

## Discussion

### Main findings of the study

By using data from a population-based study with over 15 follow-up years, we attempted to examine the relationships of reproductive factors with adiposity and body shape indices changes overtime. Our study findings showed higher BMI in females with early menarche and higher values of BMI and WC and lower values of ABSI in women with premature/early menopausal age than those with normal age at menopause. A marginal effect of parity on BMI and WC was detected as well, indicating that women with parity ≥ 3 had higher mean changes in these parameters, compared to those with less parity.

### Possible mechanisms involved in the relationship between reproductive factors, adiposity, and body shape indices

There is evidence demonstrating more prevalence of obesity and other adiposity indices in women than in men worldwide, which highlights the role of women’s reproductive factors in developing these disorders [4, 5]. Although the underlying mechanisms have not been completely elucidated yet, ovarian hormone alterations within the reproductive lifespan and menopause transition of women seem to play a major role. In this regard, estrogen has been introduced as a key factor in the causes, consequences, and distribution of fat among women. Estrogens synergize with adipose tissue genes to increase gluteofemoral subcutaneous adipose tissue mass and decrease central adipose tissue mass in reproductive-age women. Deprivation of estrogens after menopause, therefore, independent of aging, is associated with an increase in total adipose tissue mass and a decline in lean body mass. Menopause also partially reverses women’s protective adipose tissue distribution [10]. Indeed, the neuroendocrine system regulates the body fat content of the human body throughout its lifespan [40]. Accordingly, women in their different reproductive stages, such as menarche, pregnancy, postpartum, and menopause are exposed to various hormonal changes. These transitions can lead to substantial alterations in metabolic status and body structure [16].

### Age at menarche, adiposity, and body shape indices

Earlier studies have demonstrated a significant association between age at menarche and adiposity and body shape indices, although this relation has been complicated by mutual causation [41]. Obesity has been proposed as a strong risk factor for age at menarche. Some studies have reported that the typical female pattern of regional adipose tissue distribution emerges after puberty [21, 22], indicating that a normative young adult woman has ~ 18 kg body fat (~ 30% of body weight), compared to a normative young adult man who has ~ 12 kg body fat (~ 15% body weight) [21, 42]. The most likely justification for this gender variation refers to the influences of ovarian hormones and eating behaviors after menarche. Increased concentrations of estrogens or other reproductive hormones during puberty are differentially associated with the activation of the homeobox family (HOX) and other genes to determine regional adipose distribution [10]. The majority of previous studies have demonstrated an inverse association between age at menarche and adiposity indices, in particular bodyweight [6, 17, 21–27]. In this regard, data from a historical cohort of 3743 Scottish females revealed an inverse relationship between age at menarche and BMI in middle age, which was not explained by early childhood BMI. They concluded that age at menarche, as a substantial marker for the sexual maturation process in women, may lead to differences in their adiposity indices [24]. A multicenter, community-based study on 1214 black and white women has demonstrated that earlier menarche is positively associated with visceral and subcutaneous abdominal ectopic fat independent of confounders and young-adult BMI, which could be explained by weight gain between young adulthood and midlife [25]. The Framingham Heart Study has reported on the association between age at menarche and visceral and subcutaneous adipose tissue (SAT), indicating a significant association of earlier age at menarche with greater midlife visceral adipose tissue (VAT) and SAT; these associations were attenuated when the authors adjusted for midlife BMI [27]. A population-based prospective study involving 15,807 women aged 40–79 years demonstrated that early age at menarche < 12 years was associated with increased risk of cardiovascular disease events, and its related mortality, a relationship which appeared to be only partly mediated by increased adiposity [26]. On the contrary, a prospective study on 1462 Swedish women showed no signification association between age at menarche and subsequent fat distribution [43]. Finally, findings from a meta-analysis of 10 cohort studies demonstrated that early menarche (< 12 years of age) was associated with higher BMI; it indicated that the mean BMI in women who experienced early menarche was 0.34 kg m^2^ higher compared to those who experienced menarche at 12 or more years of age [23].

In agreement with previous studies, the results of the current study indicate that the mean changes in BMI in women with early menarche was 1.43 kg/m^2^ higher than those with normal menarche age, a finding that remained significant even after adjustment for confounders. On the other hand, the mean changes in BMI in women with late menarche were significantly lower than those with normal age at menarche in both models unadjusted and adjusted. While BMI had uprising trends in all groups, regardless of their menarche age, the interaction between time and different subgroups of menarche age (early and late) was not significant. We also found a marginally significant effect of menarche age on WC which remained even after adjusting for potential confounders, indicating that the mean changes of WC in women with late menarche age were lower than those with normal menarche age. We found no significant association between menarche age and ABSI overtime. The ABSI is calculated based on the values of WC, BMI, and height and allows us to estimate visceral obesity and overall adiposity. It has been shown that this indicator can predict clinical outcomes independently of BMI [44]. The lack of significant association between age at menarche and ABSI, therefore, highlights the impact of age at menarche on overall obesity but not visceral adiposity.

### Menopausal status, adiposity, and body shape indices

Evidence also indicates that estrogen deprivation during the menopause transition can be independently associated with an increase in total adipose tissue. On the other hand, hormone therapy with estrogen is related to adiposity loss [17]. Despite reducing energy expenditure in the aging period, levels of sex hormones dramatically decline during the menopausal transition, and body fat distribution alters with reproductive aging; however, accurate biologic processes involved in changes in body fat distribution and body shape during the menopausal transition have not been elucidated [16]. Some studies have demonstrated that in both normal and mildly obese women menopause increases body fat by ~ 5% of body weight and decreases fat-free body mass by a slightly smaller amount [45–47]. It has also been suggested that reproductive hormones can significantly modulate these physiological controls of eating which may be influenced by cognition alteration after menopause, although further human studies are still required to corroborate these mechanisms [48]. Despite documentation of the proposed mechanisms mentioned above, the association between age at menopause and body adiposity is still debated and the results of previous studies are conflicting [16, 17, 20, 27, 28, 49]. The Framingham Heart Study conducted on 522 women revealed no association between menopausal age and several measures of body composition, including BMI and WC [27]. Likewise, a Japanese study conducted on 1022 women found no relationship between BMI and age at natural menopause [28]. In contrast, an observational study on both populations of pre-and postmenopausal women highlights the role of the hormonal environment in creating changes related to body fatness, indicating that the rate of reproductive aging is significantly associated with the body fat pattern. In addition, the BMI of women with abnormal levels of sex hormones was more correlated with their hormonal status than their age or menopausal status [16]. A longitudinal study on 213 Czech women showed that the majority of adiposity indices like waist, WHR, hip, and subgluteal thigh circumference increase significantly in the menopausal group [49]. Another study on 300,000 adult Chinese women from 10 diverse areas showed that later age at menopause and longer reproductive years were independently associated with increased adiposity late in life [20].

Our study results indicate that mean changes of BMI overtime were significantly higher in women with premature/ early menopausal age than those with normal menopausal age, even after adjusting confounders, whereas women with late menopausal age had higher WC and ABSI values. Our study findings, therefore, suggest that having a longer reproductive life span, likely through reproductive events like pregnancy and related factors, can lead to increasing WC values overtime, the hypothesis needs to confirm by further well-design longitudinal studies.

### Parity, abortion, adiposity, and body shape index

It is well-known that pregnancy triggers weight gain and obesity in women [12] as a result of hormonal changes during pregnancy, increased dietary intake, changes in energy balance, heritable characteristics, adverse lifestyle risk factors associated with childrearing, and other postpartum behaviors [11–13]. There is also evidence estimating that multiparous women have higher BMI than their nulliparous counterparts [14]. On the other hand, women’s body shape may be influenced by their pregnancies due to the mobilization of polyunsaturated fatty acids (PUFAs) from the lower parts of their bodies to meet the needs of the developing fetus [15]. A large number of studies have assessed a link between parity and excess body weight after childbirth, and the majority of these studies reported a positive association [6, 14, 17, 18, 20, 29]. In this regard, a cross-sectional study revealed that adiposity was associated inversely with age at first birth. In addition, the mentioned study reported that there was a non-linear positive association between adiposity and parity [20]. Likewise, data from the Stockholm Pregnancy and Weight Development Study (SPAWN), after 15 years of follow-up, showed an increment of 0.5 kg body weight per pregnancy [50]. The Coronary Artery Risk Development in Young Adults (CARDIA) study with over 10 years of follow-up demonstrated greater values of WC and WHR in multiparous women compared to nulliparous women [51]. Findings from the Million Women Study indicated that childbearing patterns had a persistent effect on BMI, indicating that women with more births had higher BMI than nulliparous women. On the other hand, at every parity level, a reduction in BMI is associated with just 6 months of breastfeeding in women [18]. A prospective study on Swedish women reported similar findings, indicating that parity was positively associated with total as well as central obesity, and lactation time was positively related to abdominal fat cell diameter [43]. Findings from the Third National Health and Nutrition Examination Survey (NHANES III) demonstrated that after adjusting age and BMI, an increasing parity in women was significantly associated with a relative decrease in hip circumference and an increase in WC after controlling for age and BMI [30]. A cross-sectional survey of 4130 white British women, using three-dimensional photonic scanning showed that parous women ≤ 40 years, parity, independent of age and BMI, was associated with increased abdominal dimensions and reduced hip and thigh dimensions compared to nulliparous, independent of age and BMI, although the effects of parity on shape diluted over time [19]. This finding indicates that the effect of pregnancy is to accelerate an underlying age-associated fat redistribution in younger adult females. On the contrary, data from a cross-sectional study on 508 Chilean women revealed that BMI, but not other adiposity parameters like WC, WHR, and waist-to-height ratio (WHtR), was modestly affected by parity after controlling by individual, reproductive, and metabolic confounders, finding suggesting a little or no influence of parity on the adiposity indices, in particular central [14].

While at the initiation of the study, we expected to observe a strong association between parity and adiposity measurements, our study findings showed just a marginal effect of parity on BMI and WC, indicating that women with parity ≥ 3 had higher mean changes of these parameters, compared to those with less parity. Abortion was not significantly associated with none of the adiposity indices. However, it should be kept in mind that the reason for this weak difference and conflicting results might be explained by a lack of consideration of some potential confounders like age at first pregnancy, lactation, birth interval, etc.

## Strengths and limitations

The greatest strength of our study is its design as a community-based study with a long-term follow-up, which enables us to make causal inferences about trends of adiposity and body shape indices overtime considering reproductive factors in an unselected population. This study has also some limitations. First, while we tried to adjust all potential confounders, other risk factors like lifestyle, socioeconomic status, nutrition patterns breastfeeding patterns, and a history of medical intervention for the management of obesity could not be considered due to the unavailability of data. Second, we could not assess body composition indices since their data were not available. Finally, it should be also considered that the TLGS has a representative sample of an urban Iranian population, caution should be used when attempting to generalize findings to rural people.

## Conclusion

This study demonstrated higher BMI in females with earlier menarche age. We also showed higher values of BMI overtime in women with premature/ early menopause, whereas women with late menopausal age had higher WC and ABSI values. A marginal effect of parity on BMI and WC was detected as well, indicating that women with parity ≥ 3 had higher mean changes in these parameters, compared to those with less parity. However, more longitudinal studies investigating body composition indices by adjusting all potential confounders are still required to confirm our study findings.

## Data Availability

The datasets generated during the current study are available from the corresponding author upon reasonable request.
